# Antiviral Responses by Swine Primary Bronchoepithelial Cells Are Limited Compared to Human Bronchoepithelial Cells Following Influenza Virus Infection

**DOI:** 10.1371/journal.pone.0070251

**Published:** 2013-07-10

**Authors:** Mary J. Hauser, Daniel Dlugolenski, Marie R. Culhane, David E. Wentworth, S. Mark Tompkins, Ralph A. Tripp

**Affiliations:** 1 Department of Infectious Diseases, College of Veterinary Medicine, University of Georgia, Athens, Georgia, United States of America; 2 Department of Veterinary Population Medicine, College of Veterinary Medicine, University of Minnesota, St Paul, Minnesota, United States of America; 3 J. Craig Venter Institute, Rockville, Maryland, United States of America; Centre of Influenza Research, The University of Hong Kong, Hong Kong

## Abstract

Swine generate reassortant influenza viruses because they can be simultaneously infected with avian and human influenza; however, the features that restrict influenza reassortment in swine and human hosts are not fully understood. Type I and III interferons (IFNs) act as the first line of defense against influenza virus infection of respiratory epithelium. To determine if human and swine have different capacities to mount an antiviral response the expression of IFN and IFN-stimulated genes (ISG) in normal human bronchial epithelial (NHBE) cells and normal swine bronchial epithelial (NSBE) cells was evaluated following infection with human (H3N2), swine (H1N1), and avian (H5N3, H5N2, H5N1) influenza A viruses. Expression of IFNλ and ISGs were substantially higher in NHBE cells compared to NSBE cells following H5 avian influenza virus infection compared to human or swine influenza virus infection. This effect was associated with reduced H5 avian influenza virus replication in human cells at late times post infection. Further, RIG-I expression was lower in NSBE cells compared to NHBE cells suggesting reduced virus sensing. Together, these studies identify key differences in the antiviral response between human and swine respiratory epithelium alluding to differences that may govern influenza reassortment.

## Introduction

Influenza viruses pose a significant risk to human health due to their continuous evolution and zoonotic potential. Vaccination can prevent or reduce illness associated with seasonal influenza virus infection, however the continuing emergence of influenza strains to which the population is immunologically naïve is a threat to public health [1]. Influenza viruses are members of the *Orthomyxoviridae* family which comprise a group of enveloped, segmented, negative-strand RNA viruses. The segmented nature of the influenza viral genome allows for reassortment among virus strains which is a factor in virus adaptation [2]. Genetic drift and reassortment with avian, swine and human-derived genome segments has made a universal vaccine problematic with only seasonal protection currently afforded by the yearly vaccine [3]. Four major pandemic influenza outbreaks have occurred in the past century with the most recent occurring in 2009. The influenza pandemic of 1918 resulted in the deaths of 50 million people and based on analysis using Bayesian relaxed clock methods the virus was generated by reassortment between avian viruses and previously circulating human and swine strains over a period of years [4]. Viruses that caused the influenza pandemics of 1957 and 1968 were generated by reassortment of an avian strain with a 1918 virus descendent [5]. The ongoing risk that viral reassortment poses was highlighted by the emergence of the 2009 triple reassortant swine-origin H1N1 pandemic virus [6]. Furthermore, the highly pathogenic H5N1 avian influenza virus has recently crossed the species barrier to infect humans resulting in a high mortality rate, and reassortant viruses with internal genes of avian H5 lineage have been identified in swine, raising concern about the pandemic potential of reassortant H5 viruses [7,8].

The natural host for all influenza A viruses are wild aquatic birds, but many animal species are spill-over hosts including humans, swine, horses, and others that can be infected [9]. It has been hypothesized that swine are an intermediate host for transmission of avian viruses to humans [10]. Swine can be infected with influenza viruses of avian, swine, and human origin, reassortment among influenza viruses derived from these species can occur in swine, and resulting reassortant strains can be transmitted from swine to humans [11]. The basis why swine more readily support influenza virus reassortment than humans is not understood. Traditionally, the susceptibility of swine to both avian and human influenza viruses has been attributed to the presence of receptors for avian (α-2,3 linked sialic acid) and human (α-2,6 linked sialic acid) influenza viruses in their respiratory tract [12,13]. However, more recent studies have disputed the distribution of these sialic acid receptors in the swine respiratory tract, as well as the necessity of their presence for infection. Recent reports have shown that swine and humans have similar respiratory expression of α-2,3 and α-2,6 linked sialic acid [14,15]. Likewise, one study showed that α-2,6 linked sialic acid was the predominant receptor in all areas of the swine respiratory tract [16]. Additionally, a recent study showed that avian influenza viruses can infect and replicate in fully differentiated, primary NHBE cells independent of detectable sialic acid expression [17]. Together, this suggests that there are other features that likely contribute to influenza virus infection and reassortment in swine. Given the critical role of antiviral IFN, it is likely that host innate responses contribute to restriction of influenza virus infection and reassortment in human and swine respiratory epithelial cells following infection.

The innate immune response is the first line of defense against influenza infection. Among innate responses, type I and III IFN induction and signaling is a potent mechanism of protection against viral infection [18]. Hundreds of ISGs have been shown to be expressed following IFN signaling, which act to restrict infection by multiple mechanisms [19]. Humans and swine have been shown to induce expression of similar cytokines following *in vivo* influenza A infection, including IFNα, TNFα, and IL-6 which peak 1-2 days following infection [20–22]. However, due to varied experimental systems and a lack of swine reagents, it remains difficult to establish whether differences exist in the tempo and magnitude of the innate response between humans and swine.

To address important differences between human and swine respiratory epithelial cells’ ability to mount an antiviral response, both fully differentiated and undifferentiated primary human and swine respiratory epithelial cells were examined for type I and III interferon responses following infection with human, swine, and avian influenza viruses. Human respiratory epithelial cells had substantially higher IFNβ, IFNλ, and ISG gene expression following influenza infection compared to swine cells. Swine respiratory epithelial cells were capable of mounting an antiviral response, but it was lower in magnitude and delayed compared to human cells. Multistep influenza virus growth kinetics were analogous between human and swine with the exception of very late times post-infection where H5 avian influenza virus titers declined more rapidly in human cells compared to swine cells. Studies using an influenza virus NS1 mutant eliminated a role for viral antagonism in the differential antiviral gene expression, and reduced antiviral gene expression in swine respiratory epithelial cells was global rather than influenza-specific based on treatment of cells with synthetic dsRNA.

## Materials and Methods

### Cells and reagents

Isolation of normal swine bronchial epithelial cells was performed as previously described with modification [23,24]. Lungs from healthy, adult swine were obtained from the University of Minnesota Pre-Clinical Resource Center. Euthanasia and tissue harvest were approved by the University of Minnesota Institutional Animal Care and Use Committee, conducted in compliance with the Animal Welfare Act, and adhered to principles stated in the Guide for Care and Use of Laboratory Animals. Six healthy adult purpose bred swine were enrolled as pancreas donors as part of a preclinical islet xenotransplantation program. Animals were anesthetized with IM Telazol (Fort Dodge Laboratories, Fort Dodge, IA) for scheduled euthanasia, performed by electric stun and immediate exsanguination. Post mortem tissue was obtained via the tissue-sharing program Preclinical Research Center, Department of Surgery, at the University of Minnesota.

The swine trachea and bronchi were cut into 1 x 2cm sections and placed into digestion media composed of DMEM supplemented with 1.5 mg/ml Pronase (Roche Applied Science, Indianapolis, IN), 10 µg/ml DNase (Sigma-Aldrich, St. Louis, MO), 100 µg/ml primocin (InVivogen, San Diego, CA) and 1,000 I.U. /ml penicillin, 1,000 µg/ml streptomycin, and 2.5 µg/ml amphotericin (10x antibiotic/antimycotic Solution from CellGro, Manassas, VA). After 72 hours incubation at 4°C on a rocking platform, the luminal surface of the tissue was gently scraped with a surgical scalpel, and the cells passed through a cell strainer and centrifuged at 500g for 5 min, resuspended in DMEM and plated onto non-coated flasks for 2 hours at 37°C. To remove fibroblasts, the remaining non-adherent cells were collected and resuspended in BronchiaLife B/ T medium Complete Kit containing the same antibiotics as the digestion solution described above, and plated onto flasks pre-coated with rat tail collagen (BD Biosciences, Franklin Lake, NJ). The normal swine bronchial epithelial (NSBE) cells were expanded to 70% confluence and cryopreserved in 10% DMSO (Sigma) and 90% fetal bovine sera (Hyclone) and stored in liquid nitrogen vapor. Cells were determined to be free of influenza virus, parvovirus, pseudorabies virus, porcine reproductive and respiratory syndrome virus, circovirus type 2, and rotavirus as determined by the Athens Veterinary Diagnostic Lab.

NHBE cells from a 17 year old healthy male (Lifeline Cell Technology, Frederick, MD) were expanded and cryopreserved according to manufacturer’s instructions. To facilitate differentiation, NHBE and NSBE cells were cultured at air-liquid interface as previously described with slight modifications [16]. Briefly, cells were seeded at a density of 120,000 cells/cm^2^ onto collagen coated 0.33 cm^2^ transwell permeable supports with 0.4µM pores (Costar) and submerged under BronchiaLife B/ T medium Complete Kit (Lifeline Cell Technology, Frederick, MD) until they reached confluence. Once confluent, the apical chamber was left exposed to humidified 95% air/5% CO_2_, and a 1:1 mix of DMEM and BronchiaLife B/T containing one complete supplement kit and 50µM retinoic acid was replaced 3 times a week in the basolateral chamber until well-differentiated. Trans-epithelial electrical resistance (TEER) measurements were taken using an EVOM epithelial voltohmmeter (World Precision Instruments, Sarasota, FL) to ensure high trans-epithelial cell resistance indicating tight junction formation. Undifferentiated cells were cultured in collagen-coated plates in BronchiaLife B/ T medium Complete Kit and infected 2 days after plating.

Polyinosinic-polycytidylic acid (poly I:C) high molecular weight (Invivogen, San Diego, CA) was added exogenously to cells at a concentration of 50 µg/ml. RNA was collected or cells were infected 24 hours later. Rat tail collagen (BD Biosciences) was used at 50µM diluted in 0.02N acetic acid to coat plates and transwells.

### Viruses, infection, and growth kinetics

Human seasonal H3N2 A/New York/55/2004 was kindly provided by Richard Webby, St. Jude Children’s Research Hospital, Memphis TN. Swine H1N1 A/swine/Minnesota/02749/2009 is a primary isolate received from the University of Minnesota, St. Paul. 2009 pandemic H1N1 A/New York/1682/2009 and the corresponding NS1-126 deletion mutant virus were received from State University of New York, Albany, NY [22]. Human and swine stocks were propagated in Madin-Darby Canine Kidney (MDCK) cells in the presence of 1 µg/ml trypsin. Low pathogenic avian influenza strains A/chicken/Texas/167280-4/02 (H5N3) and A/chicken/Pennsylvania/13609/1993 (H5N2) were kindly provided by David Suarez, USDA-Southeast Poultry Research Laboratory, Athens, GA. Low pathogenic A/Mute Swan/Michigan/06/451072-2/2006 (H5N1) was kindly provided by David Stallknecht, University of Georgia, Athens, GA. Avian viral stocks were generated by inoculating 9-day old specific pathogen free (SPF) chicken eggs and harvesting the allantoic fluid 48 hours later. All viral titers were determined by plaque assay on MDCK cells as previously described [4].

Prior to infection, differentiated cells were washed 3 times with PBS to remove mucus on the apical surface. Viruses were diluted in BronchiaLife B/ T medium without supplements and applied to the apical surface of differentiated cells for 1 hour at 37°C. Virus dilutions were removed and the apical surface was washed 3 times with PBS to remove residual virus. Undifferentiated cells were infected in a similar manner but were supplemented with 1 µg/ml TPCK-trypsin. Viral titers were determined by plaque assay on MDCK cells for both differentiated and undifferentiated cells as described previously [4].

### Immune staining and Western blotting

Differentiated NHBE and NSBE cells were fixed for 30 min on the transwell with 4% formaldehyde. For lectin staining of sialic acids, cells were incubated for 1 hour with 20 µg/ml biotinylated SNA to detect α-2,6 linked sialic acids or biotinylated MAA-II (Vector Laboratories, Burlingame, CA) to detect α-2,3 linked sialic acids, then incubated with 15 µg/ml Texas Red-streptavidin (Vector Laboratories, Burlingame, CA) to visualize lectin binding. For detection of mucus secreting goblet cells and ciliated cells, the cells were permeabilized with 0.5% Triton-X 100 in PBS and incubated with a mouse anti-mucin 5AC antibody (Thermo Scientific, Kalamazoo, MI) and secondary anti-mouse IgG Alexafluor488 (Molecular Probes, Carlsbad, CA) or anti-β-tubulin directly conjugated to Cy3 (Abcam, Cambridge, MA). All antibodies were diluted in 0.05% TWEEN in PBS. Cells were rapid stained with 1 µg/ml DAPI. Transwell membranes were excised with a scalpel and mounted onto glass slides. Micrographs were taken on a Nikon A1R Confocal Microscope (Nikon Instruments Inc., Melville, NY) at 20x magnification. To confirm that cells isolated from swine lungs were of epithelial origin, cells were stained with a mouse anti-cytokeratin antibody and a secondary anti-mouse IgG Alexafluor488. Micrographs were taken on an EVOS fluorescence microscope (Advanced Microscopy Group, Bothell, WA) at 20x magnification.

To detect protein by Western blotting, cells were lysed in 1% sodium dodecyl sulfate and boiled for 5 min at 100°C. After quantification of total protein using a BCA protein assay kit (Thermo Scientific, Rockford, IL), 20µg of protein was separated on a 4-20% tris-glycine gel and blotted onto PVDF membrane. The blot was probed with a rabbit anti-human RIG-I antibody (product #4200, Cell Signaling Technology, Boston, MA) or mouse anti-human GAPDH (Millipore, Billerica, MA) followed by species specific secondary HRP conjugated antibodies. Western blots were developed with SuperSignal West Pico Chemiluminescent substrate (Thermo Scientific, Rockford IL) and visualized on a FluorChem Q System imager (Protein Simple, Santa Clara, CA).

### Quantitative RT-PCR

Total RNA was isolated using RNeasy Mini kits (Qiagen, Valencia, CA). Reverse transcription was performed using a SuperScript VILO cDNA synthesis kit (Life Technologies, Grand Island, NY). Human and swine specific Taqman gene expression assay primer/probe sets and master mix (Life Technologies, Grand Island, NY) were used to amplify and quantify human and swine IFN-α (Assay IDs: Hs00256882_s1 and Ss03394862_g1), IFN-β (Assay IDs: Hs01077958_s1 and Ss03378485_u1), IFN-λ (Assay IDs: Hs00601677_g1 and Ss03820546_u1), ISG15 (Assay IDs: Hs00601677_g1 and Ss03377462_u1), MX1 (Assay IDs: Hs00895608_m1 and Ss03393847_m1) and OAS1 (Assay IDs: Hs00242943_m1 and Ss03394660_m1) according to manufacturer’s protocols. HPRT (Assay ID: Hs01003267_m1 and Ss03388274_m1) was used as a housekeeping gene to normalize gene expression. M gene copy number was determined by performing a one-step RT-PCR reaction (Qiagen, Valencia, CA) using Universal influenza forward primer (GAC CRA TCC TGT CAC CTC TGA C), reverse primer (AGG GCA TTY TGG ACA AAK CGT CTA), and probe (FAM-TGC AGT CCT CGC TCA CTG GGC ACG-BHQ1) (Biosearch Technologies, Novato, CA) and values were determined by running a standard curve. All RT-PCR reactions were performed using a Stratagene Mx3005P QPCR System.

### Statistical analysis of data

Differences between human and swine antiviral gene expression and viral titers were evaluated by two-way ANOVA and a post-hoc Bonferroni test and considered significant when *p*<0.05. All data are shown as the mean *±* standard deviation.

## Results

### Isolation and characterization of NSBE cells

Primary NSBE were isolated and prepared for comparison to primary NHBE cells. NSBE isolated from the trachea and bronchi of healthy, adult swine were minimally passaged and evaluated by phase contrast and keratin staining (Figure S1A). NHBE and NSBE cells were similarly cultured at air-liquid interface using a transwell system to allow for cellular differentiation. Both cell types reached high trans-epithelial resistance, which occurred sooner in swine cells than in human cells, and cellular composition between NHBE and NSBE cells was determined to be similar by immune staining for goblet, mucus-secreting cells and ciliated cells (Figure S1B and C). Additionally, both NHBE and NSBE differentiated cells displayed predominantly α-2,6 linked sialic acid expression which is consistent with a previous finding that showed that α-2,6 linked sialic acid was the predominant receptor in all areas of the swine respiratory tract (16).

### IFN and IFN-stimulated gene (ISG) expression following influenza infection in differentiated NHBE and NSBE cells

**Figure 1:**
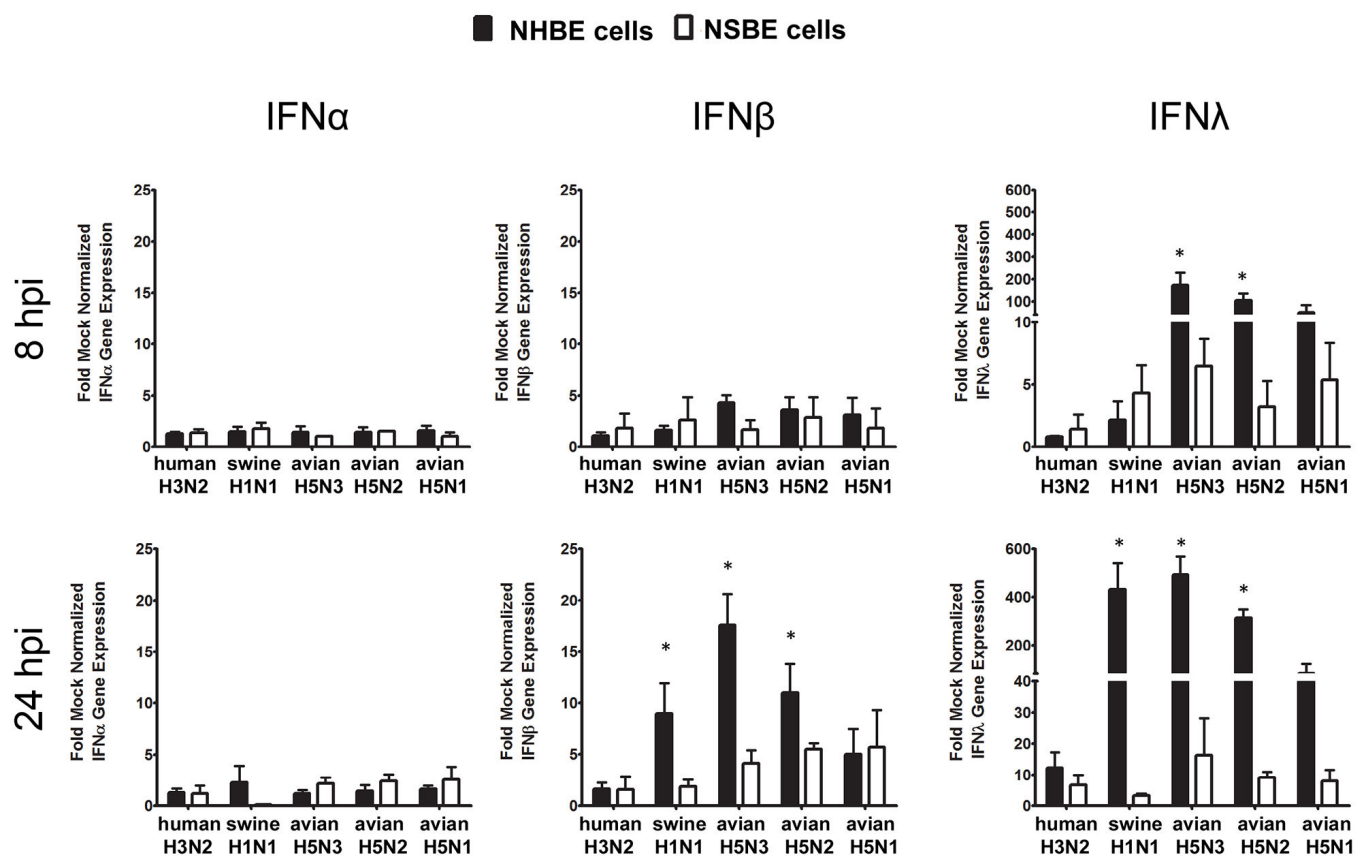
Type I and III IFN expression in differentiated NHBE and NSBE cells. NHBE (black bars) or NSBE (white bars) cells that reached a well-differentiated state were infected at a MOI of 0.01 with the indicated viruses. At 8 (top panel) and 24 (bottom panel) HPI RNA was harvested and levels of type I and III IFN genes were quantified by qRT-PCR. Results are expressed as fold over mock infected cells and normalized to a housekeeping gene. Significant differences in gene expression between human and swine cells are indicated by * and determined as described in the Material and Methods.

To determine if IFN gene expression differed between influenza-infected NHBE or NSBE cells, the two cell types were infected at a low multiplicity of infection (MOI) of 0.01 with human (A/NY/55/2004, H3N2), swine (A/swine/MN/02749/2009, H1N1), or avian influenza viruses (A/chicken/TX/167280-04/2002, H5N3; A/chicken/PA/13609/1993, H5N2; A/mute swan/MI/451072-2/2006, H5N1) and IFNα, IFNβ, and IFNλ gene expression determined at 8 and 24 hours post-infection (HPI) (Figure 1). No substantial differences in IFNα gene expression in NHBE or NSBE cells were evident at 8 or 24 HPI following infection with any virus. IFNβ gene expression was low in NHBE and NSBE cells 8 HPI with human, swine, and avian influenza virus (AIV) infections; however at 24 HPI, IFNβ expression was 5 to 20-fold higher than mock infection in NHBE cells but not in NSBE cells infected with swine and AIV. In contrast, at 8 HPI, IFNλ gene expression was considerably higher than type I IFN gene expression in both NHBE and NSBE cells infected with AIVs, and trending higher for cells infected with swine H1N1. The greatest differences in IFNλ gene expression occurred in NHBE cells infected with H5 AIV suggesting human cells respond more potently than swine respiratory epithelial cells at early time-points post-infection. Intriguingly, at 24 HPI, there was nearly a 500-fold induction of IFNλ gene expression in NHBE cells infected with AIV and swine H1N1 viruses over mock infection, and nearly a 10-fold increase in both NHBE and NSBE cells infected with human H3N2 (Figure 1). No substantial increase in IFNλ gene expression was evident in similarly infected NSBE cells. These findings show that human respiratory epithelial cells respond more robustly to H5 AIV and swine influenza virus infection compared to swine respiratory epithelial cells. In addition, the tempo of IFN gene induction also appears to be delayed in swine cells as compared to human cells.

**Figure 2:**
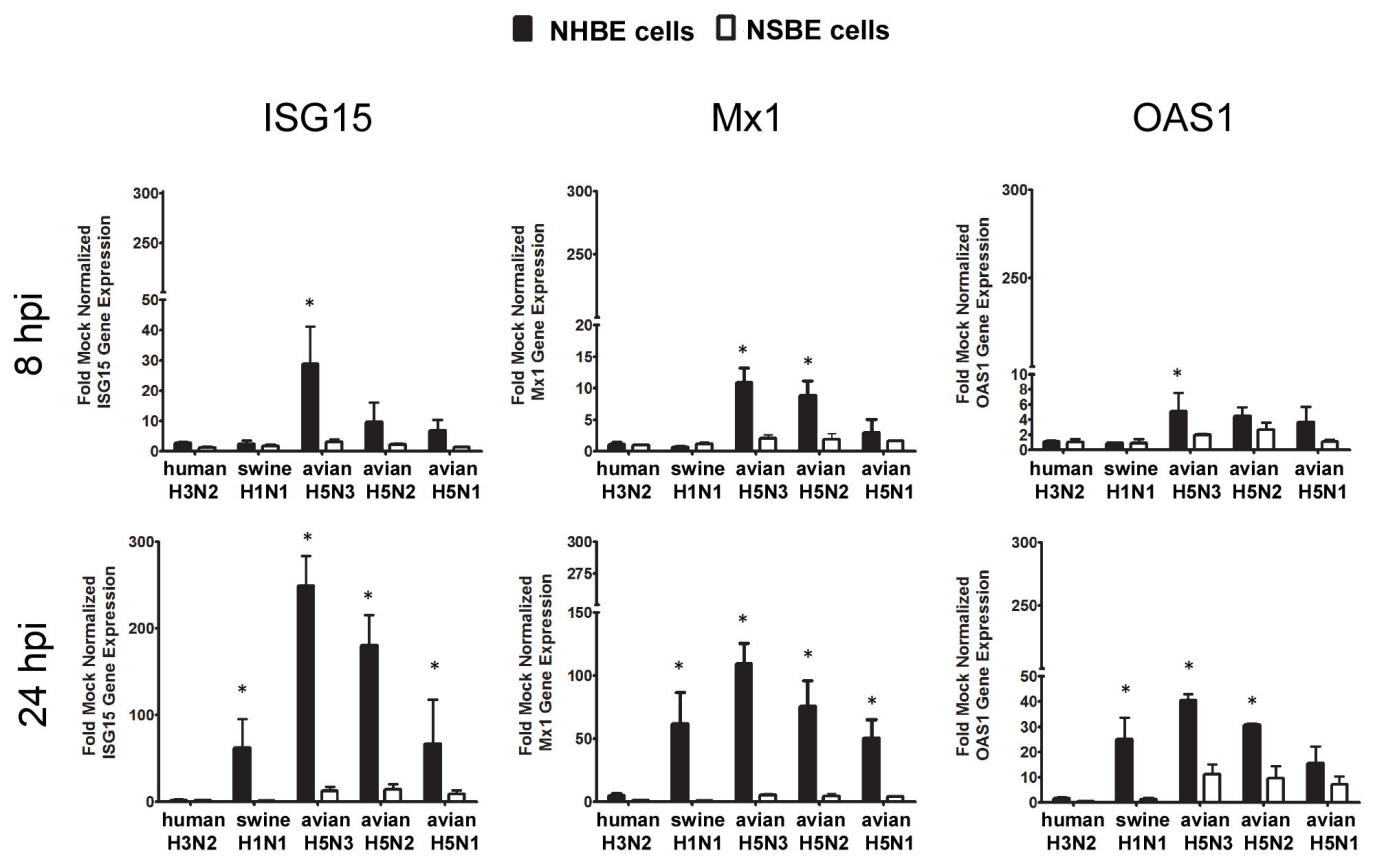
ISG expression in differentiated NHBE and NSBE cells. NHBE (black bars) or NSBE (white bars) cells that reached a well-differentiated state were infected at a MOI of 0.01 with the indicated viruses. At 8 (top panel) and 24 (bottom panel) HPI RNA was harvested and levels of the indicated ISGs were quantified by qRT-PCR. Results are expressed as fold over mock infected cells and normalized to a housekeeping gene. Significant differences in gene expression between human and swine cells are indicated by * and determined as described in the Material and Methods.

It is well-understood that IFN expression affects ISGs and that IFNβ expression governs ISG15, Mx1 and OAS1 gene expression [25,26]. Thus, to evaluate this relationship in human and swine respiratory epithelial cells, NHBE and NSBE cells were infected with human, swine and AIV at a MOI of 0.01, and expression of IFN-stimulated genes ISG15, Mx1, and OAS1 was examined (Figure 2). NHBE cells infected with AIVs, and in particular H5N3, expressed higher levels of ISG15, Mx1 and OAS at 8 HPI compared to NSBE cells whose expression was negligible. At 24 HPI, ISG15, Mx1 and OAS1 gene expression levels were markedly higher in AIV-infected NHBE cells compared to NSBE cells, a finding consistent with IFNβ gene expression (Figure 1). Also, NHBE cells infected with swine H1N1 had a substantially higher level of ISG15, Mx1 and OAS1 gene expression compared to NSBE cells. These findings show that the antiviral response in human respiratory epithelial cells occurs more rapidly and robustly than swine respiratory epithelial cells.

### Antiviral gene expression in undifferentiated NHBE and NSBE cells

**Figure 3:**
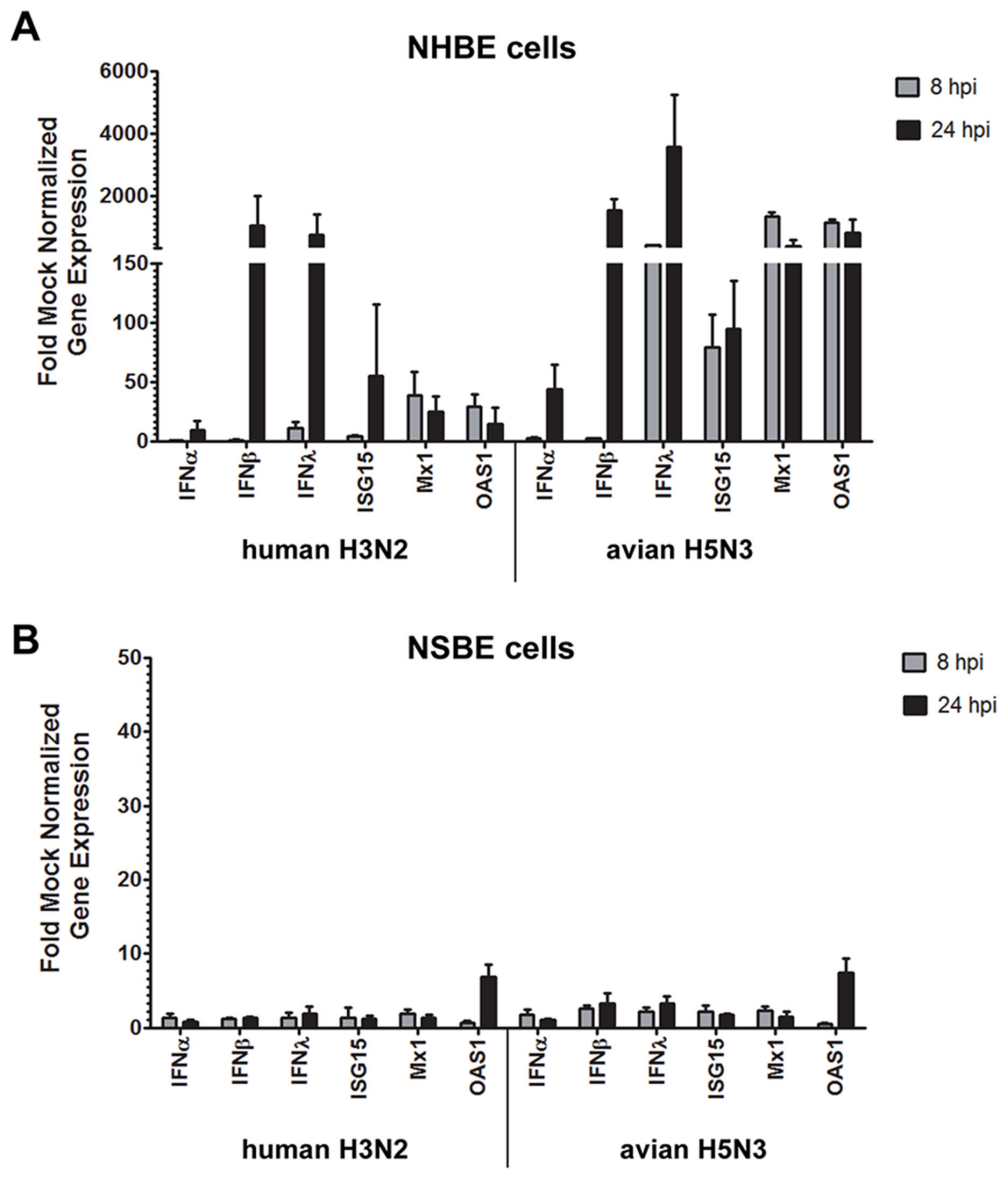
IFN and ISG expression in undifferentiated NHBE and NSBE cells. NHBE (A) or NSBE (B) cells remaining in an undifferentiated state were infected at a MOI of 0.01 with the indicated viruses. At 8 and 24 HPI RNA was harvested and levels of type I and III IFN genes and ISGs were quantified by qRT-PCR. Results are expressed as fold over mock infected cells and normalized to a housekeeping gene.

As undifferentiated cells underlie differentiated respiratory epithelial cells in the airways [27], it is important to evaluate the antiviral response to influenza virus infection of this cell type. Given the substantial differences in the antiviral responses observed between fully differentiated NHBE and NSBE cells following AIV infection (Figures 1 and 2), similar studies were performed using undifferentiated NHBE and NSBE cells infected with human H3N2 or avian H5N3 viruses (Figure 3A). These viruses were chosen because in differentiated cell cultures the human virus caused little antiviral gene induction, while the avian H5N3 virus induced robust expression of antiviral genes. At 8 HPI, undifferentiated NHBE cells infected with human H3N2 expressed elevated levels of IFNλ, ISG15, Mx1 and OAS1, but no type I IFN expression was detected. However, at 24 HPI, both IFNα and IFNβ were expressed, with high levels of both IFNβ and IFNλ gene expression evident, increased ISG15 gene expression, and continued OAS1 gene expression. In contrast, at 8 HPI, undifferentiated NHBE cells infected with avian H5N3 expressed III IFNs, ISG15, Mx1 and OAS1. By 24 HPI, avian H5N3 infected NHBE cells had similar levels of Type I and III IFNs as cells infected with human H3N2, whereas the level of ISGs induced by AIV infection remained higher (Figure 3A). Remarkably, the IFNβ and IFNλ response was 1000-fold higher in NHBE cells infected with either virus compared to mock infected cells at 24 HPI.

In undifferentiated NSBE cells, induction of IFNs and ISGs was low, reaching less than 3-fold over mock infected cells, with the exception of OAS1 at 24 HPI (Figure 3B). NSBE cells infected with avian H5N3 had marginally higher levels of antiviral gene expression; however, these differences were not significant, and again OAS1 gene expression was only slightly increased. Previous studies have also shown that OAS1 is up-regulated more readily than other ISGs following viral infection in swine cells [28]. These findings strengthen the notion that human respiratory epithelial cells respond more vigorously than swine respiratory epithelial to influenza virus infection.

### Viral growth kinetics in NHBE and NSBE cells

**Figure 4:**
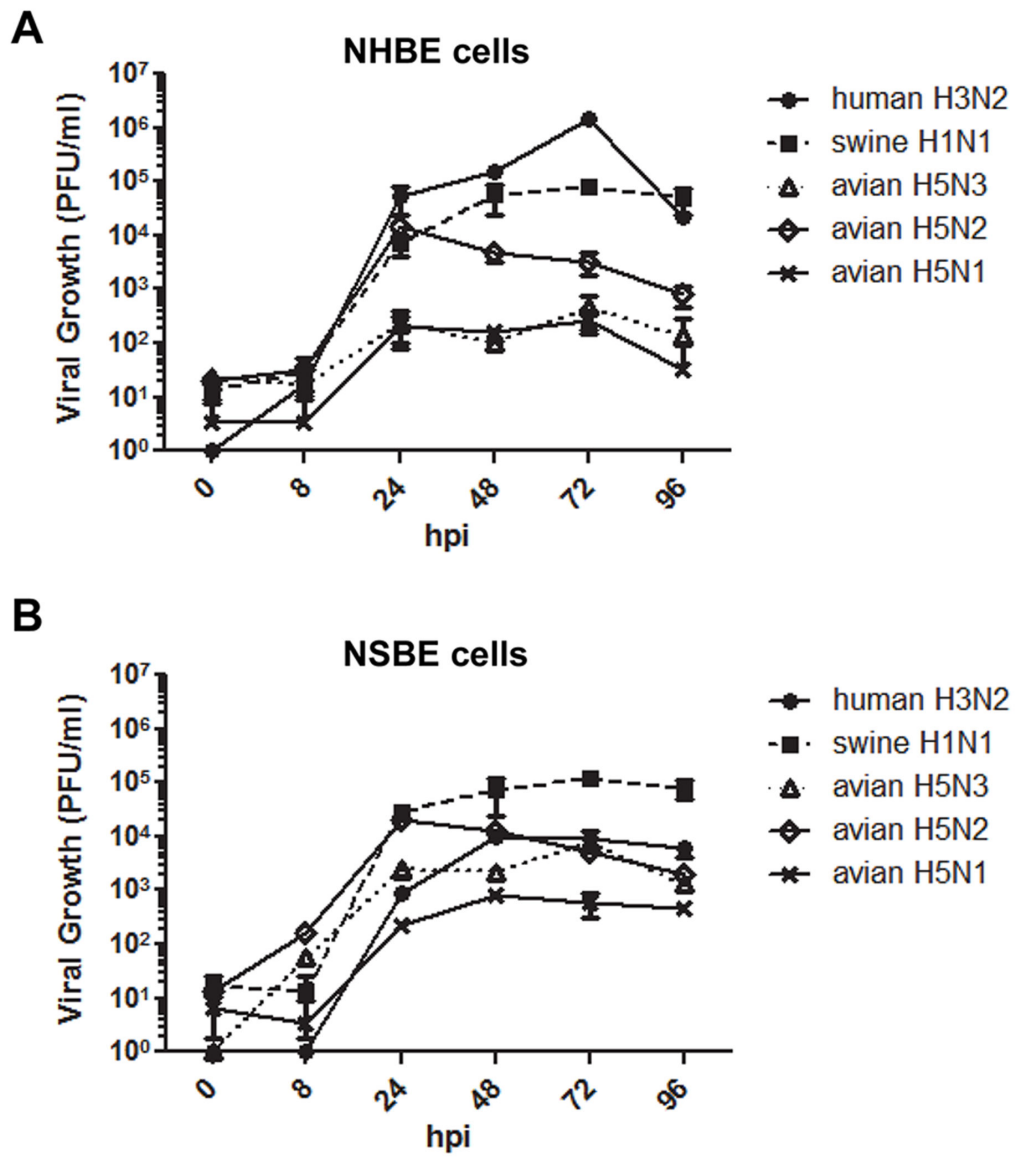
Multi-step viral growth in NHBE and NSBE cells. Undifferentiated NHBE (A) and NSBE (B) cells were infected with the indicated viruses at a MOI of 0.01. At 0, 8, 24, 48, 72, and 96 HPI supernatant was collected and viral titers were determined by plaque assay on MDCK cells.

IFNs and ISGs are known to limit viral replication, thus multi-step viral growth kinetics were assessed in NHBE and NSBE cells. Cells were infected with human H3N2, swine H1N1, or AIVs at a MOI of 0.01 and supernatants were collected from 0 to 96 HPI to determine viral titers by standard plaque assay (Figure 4). At 8 HPI, there was no difference in human, swine or AIV replication in NHBE cells, however at 24 HPI, human H3N2, swine H1N1 and avian H5N2 viruses replicated to 2 logs higher titer (10^4^ -10^5^) than avian H5N3 or H5N1 viruses (10^2^). Peak virus titers of human H3N2 and swine H1N1 viruses were 10^6^ and 10^5^, respectively, occurring at 72 HPI (Figure 4A). The peak avian H5N2 virus was 10^4^ at 24 HPI, but titers decreased to 10^3^ at 96 HPI, and peak avian H5N3 and H5N1 titers were similar (10^2^) occurring at 72HPI. Swine H1N1 virus replicated the most robustly in NSBE having the highest peak titers (10^5^) at 72 HPI (Figure 4B). Avian H5N2 grew to titers of 10^4^ by 24 HPI, while human influenza virus reached a peak titer of 10^4^ at 48 HPI. Avian H5N3 and H5N1 replicated similarly throughout the time-course. These results reflect virus tropism where human H3N2 replicates best in human respiratory epithelial cells and swine H1N1 replicates best in swine respiratory epithelial cells, and that H5 AIVs generally replicate to a lower level in human cells (10^2^) compared to swine cells (10^3^-10^4^), a finding consistent with the more robust antiviral responses by NHBE compared to NSBE cells (Figures 1-3).

### The diminished antiviral IFN response in swine cells is not mediated by influenza NS1

**Figure 5:**
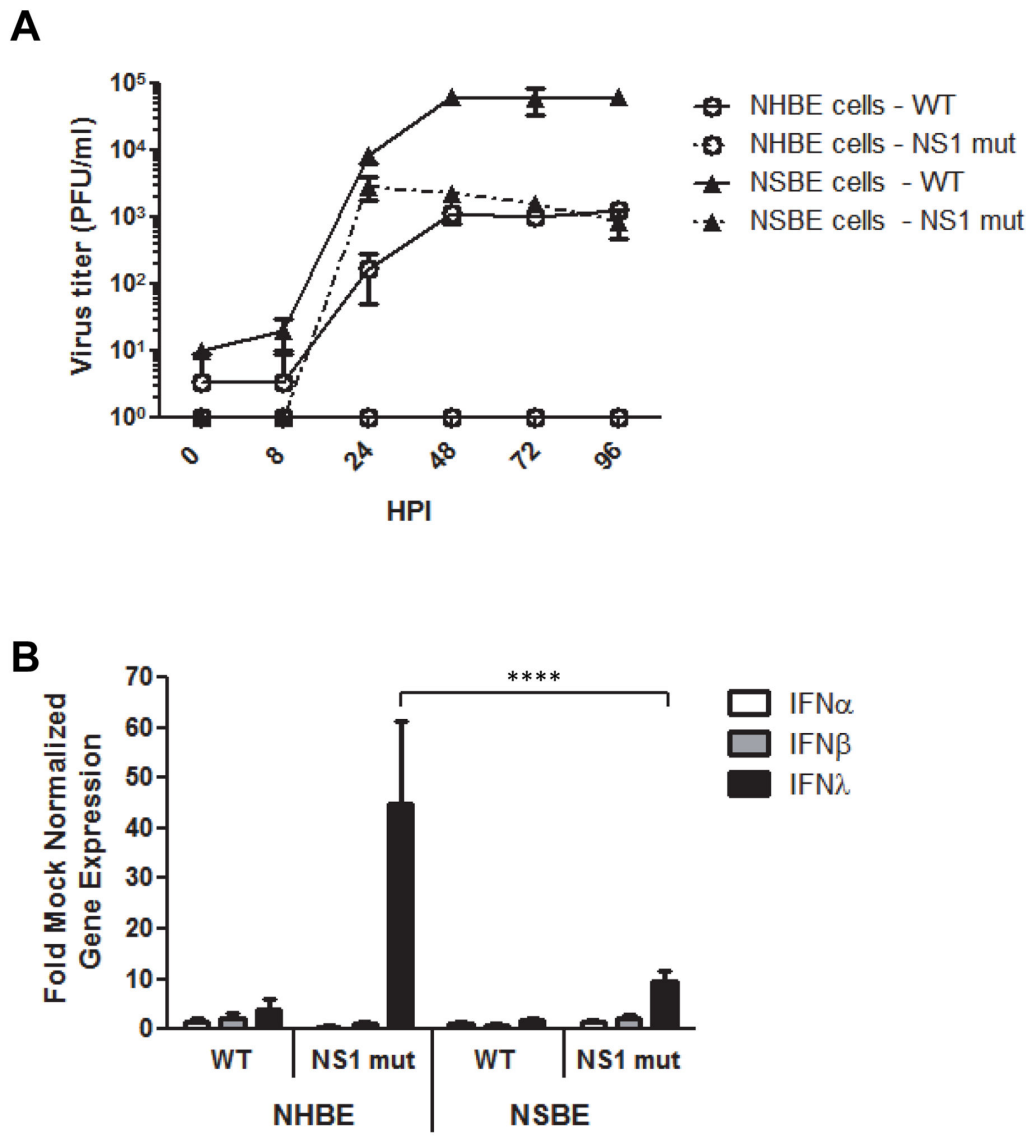
NS1 mutant replication and IFN induction in NHBE and NSBE cells. A) Un-differentiated NHBE (open circles) and NSBE (closed triangles) cells were infected at a MOI of 0.01 with A/NY/1682/2009, WT, (solid lines) or A/NY/1682/2009 NS1-126 deletion mutant, NS mut, (dashed lines). Supernatant was collected at 0, 8, 24, 48, 72, and 96 HPI and viral titers were determined by plaque assay. B) Undifferentiated NHBE and NSBE cells were infected as described in (A) and RNA was collected at 24 HPI and analyzed for type I and III IFNs by qRT-PCR. Results are expressed as fold over mock infected cells and normalized to a housekeeping gene. Significant differences in gene expression between NHBE and NSBE cells are indicated by **** where p<0.0001 and were determined as described in the Material and Methods.

There are several possible mechanisms that may contribute to the lower IFN antiviral response observed in NSBE cells compared to NHBE cells. One may be attributed to a lower level of viral replication stimulating antiviral responses in swine cells. Another may be linked to an increased ability of influenza non-structural protein 1 (NS1) to antagonize IFN pathways in swine respiratory epithelial cells. However, as H5 AIVs are able to replicate efficiently in swine cells, and at times to higher levels than that observed in human cells (Figure 4), it is less likely the level of virus replication affects antiviral responses. To determine the role of NS1 and IFN antagonism, virus titers and the level of IFN expression in NSBE and NHBE cells was determined at 0-96 HPI following infection (MOI = 0.01) of undifferentiated NSBE or NHBE cells with wild type (WT) or NS1 deletion mutant (NS1 mut) influenza viruses (Figure 5). As expected, the NS1 mutant influenza virus which lacks a functional IFN antagonist is impaired in a multi-step growth curve in IFN competent cells (46). The NS1 mutant virus replicated to 10^3^ in NSBE cells; however, no replication was evident following infection in NHBE cells compared to the WT virus which replicated to peak titers of 10^5^ in NSBE cells and 10^3^ in NHBE cells (Figure 5A). The ability of the NS1 mutant virus to replicate in swine respiratory epithelial cells, but not human, is consistent with low levels of IFN and IFN-stimulated gene expression in NSBE cells.

To specifically address induction of IFN genes, type I and III IFN expression was examined in NHBE and NSBE cells infected with WT or NS1 mut viruses (Figure 5B). If viral antagonism by NS1 was contributing to differences in IFN gene expression between human and swine cells, similar levels of IFN gene expression would be expected between the two cell types in NS1 mut infection. In the absence of a functional IFN antagonist, NHBE cells infected with NS1 mut expressed nearly 40-fold more IFNλ gene expression compared to WT infected NHBE cells with no significant effect on type I IFN expression. NSBE cells infected with NS1 mut also expressed higher IFNλ gene expression compared to WT infected cells, and there was no detectable difference in type I IFN gene expression. Interestingly, NHBE cells infected with NS1 mut had a substantial and significant (p<0.0001) increase in IFNλ gene expression compared to NSBE cells (Figure 5B). Together, this data indicates that NS1 IFN antagonism is not the primary cause for differences observed in the antiviral IFN response between swine and human respiratory epithelial cells.

### Globally diminished IFN responses in swine respiratory cells

**Figure 6:**
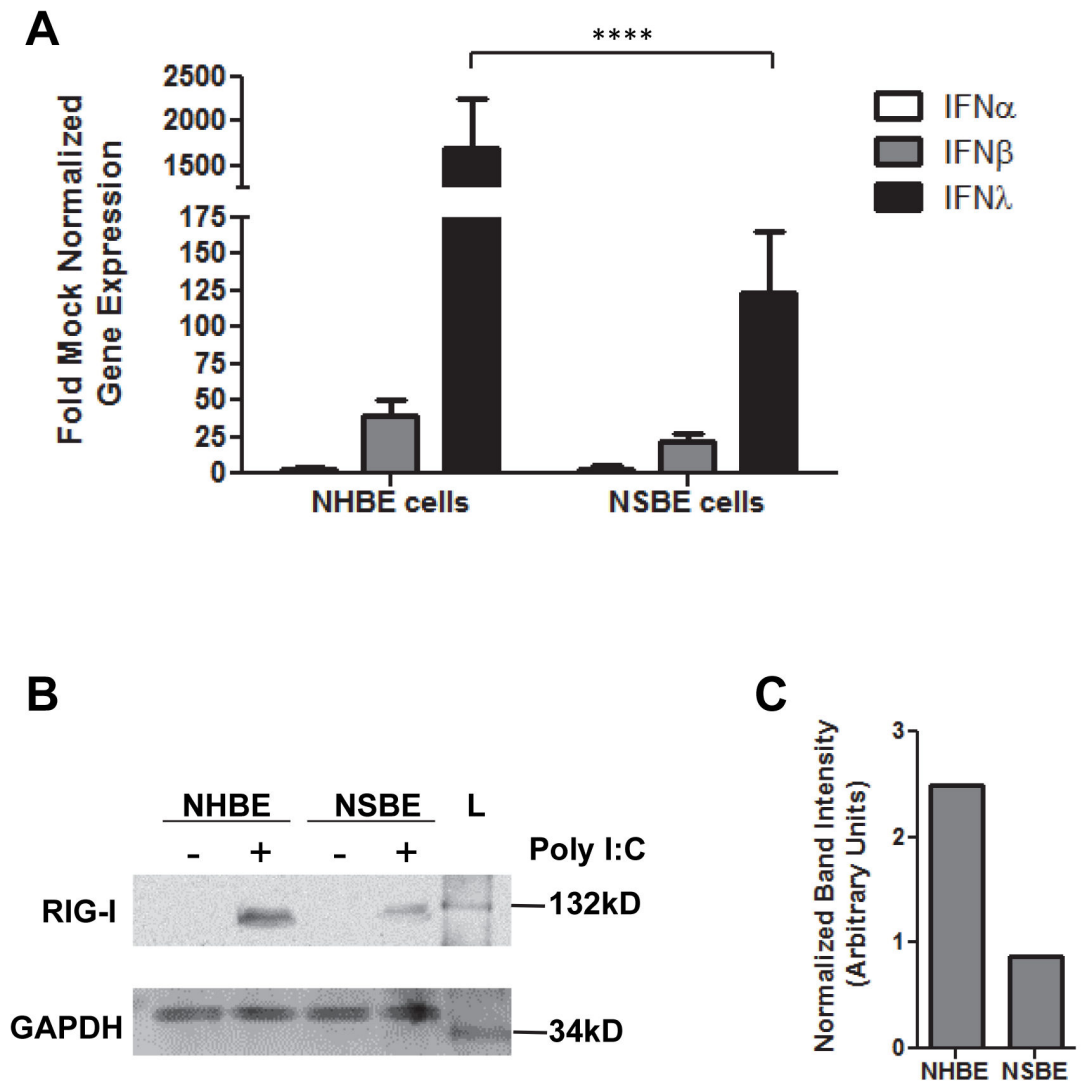
IFN gene expression following dsRNA treatment of NHBE and NSBE cells. A) Undifferentiated NHBE and NSBE cells were left untreated or treated exogenously with 50 µg/ml poly I:C. RNA was harvested 24 hours later and analyzed by qRT-PCR for IFN-α -β, or -λ expression. Results are expressed as fold over mock infected cells and normalized to a housekeeping gene. Significant differences in gene expression between NHBE and NSBE cells are indicated by **** where p<0.0001 and were determined as described in the Material and Methods. B) Cells were treated as described in (A) and 24 hours following poly I:C treatment cell lysates were harvested and subjected to Western blotting using an anti-RIG-I or anti-GAPDH antibodies. C) Quantification of band intensity shown in (B) where intensity of the RIG-I band is normalized to that of GAPDH.

The findings in this study showing that IFN-related antiviral responses in swine respiratory epithelial cells are diminished compared to human respiratory epithelial cells (Figures 1–5) raised the question of whether the lower IFN gene expression was a global response or specific to influenza virus. To address this, undifferentiated NHBE and NSBE cells were treated with a synthetic stimulator of IFNs, i.e. dsRNA or poly I:C, and 24 hours later the cell types were analyzed for expression of type I and III IFN genes. Both NHBE and NSBE cells responded to dsRNA treatment, but NHBE cells expressed a significantly (p<0.001) and dramatically higher level of IFNλ gene expression compared to NSBE cells (Figure 6A). This finding shows that swine respiratory epithelial cells have an inherently reduced capacity to generate an IFNλ response relative to human respiratory epithelial cells.

A key pattern recognition receptor that activates IFNs during influenza virus infection is RIG-I (retinoic acid-inducible gene 1) [29], thus the level of endogenous and dsRNA-induced RIG-I were determined in human and swine respiratory epithelial cells (Figure 6B). Basal levels of RIG-I could not be detected in unstimulated NSBE or NHBE cells; however, when cells were treated with dsRNA, both cell types showed an increase in RIG-I protein expression. A higher level of RIG-I was detected in stimulated NHBE cells compared to NSBE cells (Figure 6C), which likely contributes to increased amplification of IFN responses in human cells.

### Swine cells have a reduced capacity to establish an antiviral state compared to human cells

**Figure 7:**
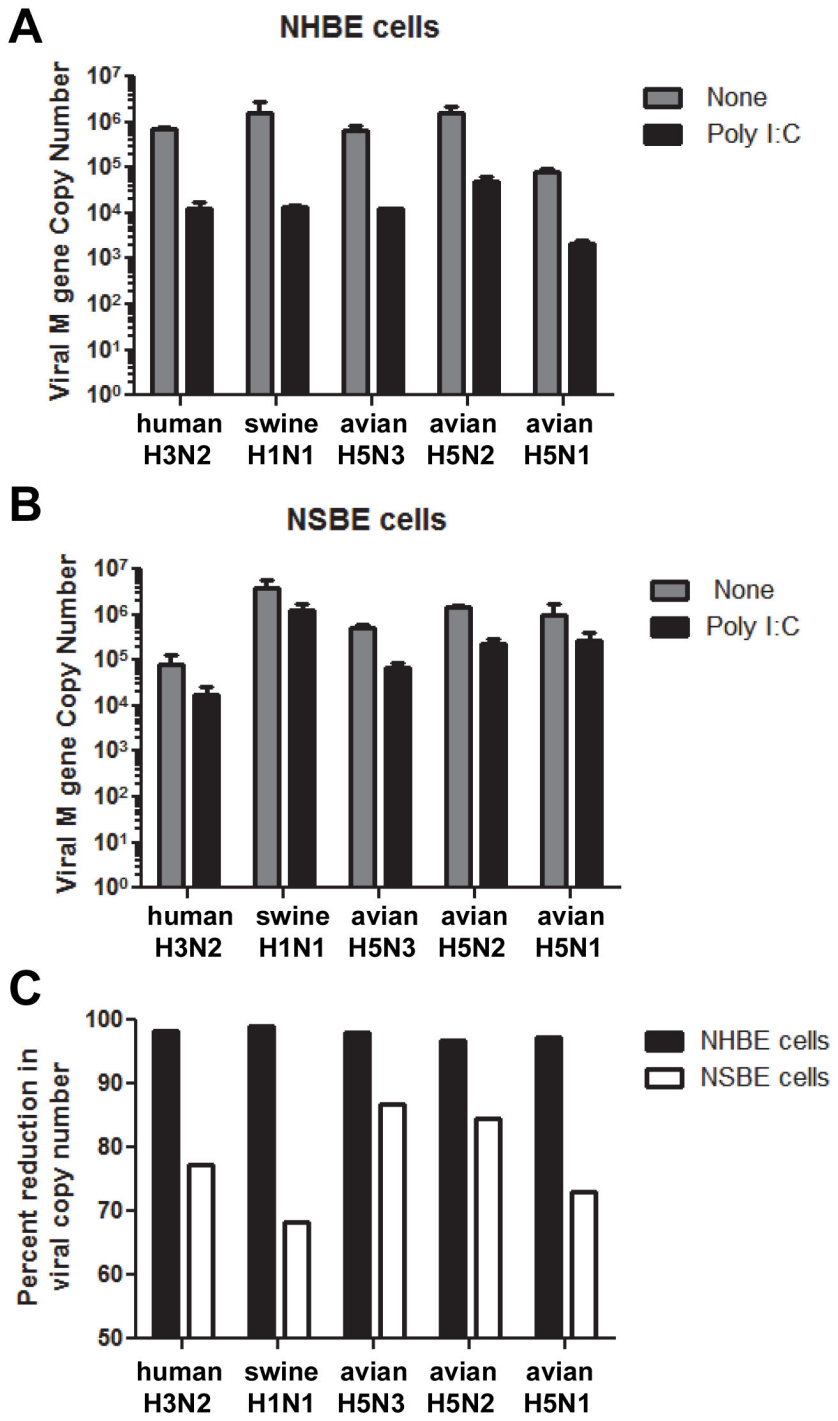
Inhibition of viral replication in cells pretreated with dsRNA. Undifferentiated NHBE (A) or NSBE (B) cells were left untreated or treated with 50 µg/ml poly I:C for 24 hours. Cells were then infected with the indicated viruses and 24 hours later the RNA was collected and evaluated for M gene copy number by qRT-PCR. C) The percent reduction in viral copy number using the values from (A) and (B) was determined and compared between human (black bars) and swine (white bars) cells.

To determine if non-specific induction of IFNs by poly I:C could induce an antiviral state in swine respiratory epithelial cells thereby making the cells refractory to influenza virus infection and replication, undifferentiated NHBE and NSBE cells were left untreated or stimulated with poly I:C and 24 hours later were infected (MOI = 0.01) with human, swine or AIVs (Figure 7). NHBE cells pretreated with the IFN-inducer, poly I:C, had viral copy numbers approximately 2 logs lower for all viruses compared to untreated NHBE cells (Figure 7A). In contrast, NSBE cells pretreated with poly I:C had virus titers that were reduced approximately 1 log for all viruses tested (Figure 7B). To clarify these findings, the percent reduction in viral copy number is given for NHBE and NSBE cells where it is clear that swine respiratory epithelial cells have a reduced capacity to enter an antiviral state compared to human respiratory epithelial cells (Figure 7C). Together, this data supports earlier findings from this study showing that human respiratory epithelial cells can more effectively express IFN-driven antiviral responses compared to swine respiratory epithelial cells.

## Discussion

Influenza viruses are highly infectious and have the ability to replicate in a broad range of host species. Avian species are the native reservoir for influenza A viruses and novel strains can be transmitted from birds to humans through direct or indirect mechanisms [30]. Swine can act as an efficient intermediate host for avian and human influenza viruses, and this is one way in which reassortant influenza viruses have the potential to create human pandemic outbreaks [31]. Thus, it is important to understand factors that may contribute to the generation of influenza reassortants in swine. To determine if the host antiviral IFN response differed between human and swine respiratory epithelial cells, responses to human, swine and AIVs were determined in primary, fully differentiated and undifferentiated cell types. The findings clearly showed that NSBE cells have a lower level of antiviral gene expression following influenza virus infection compared to NHBE cells. These findings suggest that the lowered IFN response and antiviral state of swine respiratory epithelial cells may be a key factor that allows efficient reassortment of influenza A viruses of human, swine and avian origin.

It has been known for some time that AIVs do not easily infect and replicate in humans, whereas such species barrier is lower for swine [18,32]. Transmission of AIVs to swine appears to occur regularly. Examples include detection of AIV H1N1 in European swine, and in swine in China, and several AIV strains including H4N6, H1N1, and H3N3 have been isolated from Canadian swine herds [30,33]. The capacity of some of these avian strains to become well-established in swine demonstrates their ability to successfully replicate in this species. There have been several confirmed cases of AIV infection in humans, including the highly pathogenic H5N1 AIV, however these infections have not resulted in any significant human-to-human transmission [34]. The known reduced ability of AIVs to replicate in humans was emulated in this study where virus titers in NHBE cells were significantly reduced compared to NSBE cells at late times following infection. Despite the differences in IFN and IFN-related antiviral responses between human and swine respiratory epithelial cells observed in this study, it is likely that other factors contribute to the relative differences in H5 AIV replication including levels of polymerase activity [35], and functionality of the IFN antagonist, NS1 [36].

Induction and signaling leading to the expression of type I and III IFNs is linked to the first line of defense against viral infection. IFNβ and IFNλ1, which contain similar promoter elements, are known to be the first IFNs up-regulated in response to pattern recognition receptor signaling, while IFNα gene expression is driven as part of IFN signaling amplification [37,38]. Interestingly, in this study, there was little up-regulation IFNα in both human and swine respiratory epithelial cells. This is consistent with other reports showing little to no IFNα gene expression in human airway epithelial cells following infection with negative-strand RNA viruses [39,40]. *In vivo*, IFNα is known to be an abundant and important cytokine during influenza infection, but most often this finding is linked to its expression in the serum [21]. This is expected as IFNα is expressed by a variety of cell types other than epithelial cells of the airway [41]. For example, plasmacytoid dendritic cells, which can be found in the lung as well as secondary lymphoid organs, have been shown to elicit large amounts of IFNα in response to virus infection [11].

Evaluation of the kinetics of IFN induction following influenza infection in human and swine respiratory epithelial cells demonstrated that not only was IFNλ the first IFN produced, but it was also the most abundant. Up-regulation of IFNλ before IFNβ has been observed in bronchial epithelial cells in an undifferentiated state [42], and several studies have shown that it primarily acts on epithelial cells [43,44]. Additionally, the studies reported here reveal that IFNλ gene expression occurs earlier following AIV infection compared to human and swine influenza infection in both primary NHBE and NSBE cells. Studies in A549 cells comparing AIV to the pandemic H1N1 virus report similar findings [45]. Importantly, we show evidence of species-specific influenza virus differences in antiviral gene induction in human and swine respiratory epithelial cells. This is likely a result of the balance between antiviral induction and antagonism which is determined through that action of multiple viral proteins such as those that make up the polymerase which contribute to production of pathogen associated molecular patterns (PAMPs) that activate antiviral responses as well as viral proteins like the NS1 antagonist of antiviral responses [46–48]. Other studies have shown that AIV replication in swine tracheal explants was limited compared to human and swine viruses [15]. Likewise, De Vleeschauwer and colleagues found that in pigs, an H5N2 AIV could establish an infection but produced lower virus titers and fewer antigen positive cells than a swine H1N1 virus [49]. However, human volunteers were largely refractory to AIV infection [50]. These reports highlight the need for AIV to undergo genetic changes in order to become established within the human or swine population.

The lower IFN antiviral response in NSBE cells appears not to be specific to influenza infection, but rather a global defect in IFNλ gene expression. Induction of type I and III IFNs is stimulated through recognition of foreign PAMPs by cellular sensors, including RIG-I like receptors and toll-like receptors (TLRs). Our data demonstrate that NHBE cells have more abundant expression of RIG-I than NSBE cells following dsRNA stimulation. Exogenous treatment of cells with synthetic dsRNA stimulates TLR3 activation [51] and RIG-I has been shown to be up-regulated via TLR3 signaling [52,53]. Expression of RIG-I has also been shown to be heightened following infection with Japanese encephalovirus [54], Dengue virus [55], as well as influenza virus [56,57]. Increased expression of RIG-I will serve to amplify IFN induction and signaling [58]. Thus, our data suggest that abundant expression of IFNs early following H5 AIV infection in NHBE cells leads to up-regulation of RIG-I and further amplification of the IFN response. Differential IFN induction between human and swine may be occurring at multiple levels. Here we demonstrate lower levels of RIG-I involved in the IFN induction/amplification, and others have recently reported that primary swine respiratory cells have higher levels of endogenous SOCS3 than human cells, a negative regulator of IFN signaling [59].

The implications of the findings reported here help to provide an explanation for why influenza viruses may readily infect, replicate and, in some cases, undergo reassortment in swine respiratory epithelial cells but not human respiratory epithelial cells. The tempo and magnitude of type I and III IFNs may affect the outcome of influenza virus replication and perhaps influence the frequency of reassortment during co-infection, as well as affect the gene constellation adopted by reassortant strains. These studies provide a framework for understanding influenza reassortment in swine versus human airway epithelial cells, and offer insight into disease intervention strategies to control influenza virus.

## Supporting Information

Figure S1 Characterization of differentiated NSBE cells. (A) Phase micrograph of primary swine cells (right) and fluorescent microscopy (left) showing cytokeratin staining (green) and DAPI (blue). (B) Trans-epithelial resistance of differentiated human and swine cells was measured over the indicated times post air-liquid interface (ALI). (C) Differentiated NHBE and NSBE cells were immune stained to show composition of goblet cells (top left), ciliated cells (top right), α-2,6 linked sialic acid (bottom left) and α-2,3 linked sialic acid (bottom right) expression. Cells were counterstained with DAPI (blue).
(TIF)

## References

[1] Robertson JS, Inglis SC (2011) Prospects for controlling future pandemics of influenza. Virus Res 162: 39-46. doi:10.1016/j.virusres.2011.09.024. PubMed: 21963676. 21963676 10.1016/j.virusres.2011.09.024

[2] Trifonov V, Khiabanian H, Rabadan R (2009) Geographic Dependence, Surveillance, and Origins of the 2009 Influenza A (H1N1) Virus. N Engl J Med 361: 115-119. doi:10.1056/NEJMp0904572. PubMed: 19474418. 19474418 10.1056/NEJMp0904572

[3] Carrat F, Flahault A (2007) Influenza vaccine: The challenge of antigenic drift. Vaccine 25: 6852-6862. doi:10.1016/j.vaccine.2007.07.027. PubMed: 17719149. 17719149 10.1016/j.vaccine.2007.07.027

[4] Smith GJD, Bahl J, Vijaykrishna D, Zhang J, Poon LLM et al. (2009) Dating the emergence of pandemic influenza viruses. Proc Natl Acad Sci USA 106: 11709-11712. doi:10.1073/pnas.0904991106. PubMed: 19597152. 19597152 10.1073/pnas.0904991106 PMC2709671

[5] Scholtissek C, Rohde W, Von Hoyningen V, Rott R (1978) On the origin of the human influenza virus subtypes H2N2 and H3N2. Virology 87: 13-20. doi:10.1016/0042-6822(78)90153-8. PubMed: 664248. 664248 10.1016/0042-6822(78)90153-8

[6] Morens DM, Taubenberger JK, Fauci AS (2010) The 2009 H1N1 Pandemic Influenza Virus: What Next? mBio: e00211-00210-e00211-00215. p. 1. 10.1128/mBio.00211-10 PMC2945198 20877580

[7] Cong Y, Wang G, Guan Z, Chang S, Zhang Q et al. (2010) Reassortant between human-Like H3N2 and avian H5 subtype influenza A viruses in pigs: a potential public health risk. PLOS ONE 5: e12591. doi:10.1371/journal.pone.0012591. PubMed: 20830295. 20830295 10.1371/journal.pone.0012591 PMC2935369

[8] Kalthoff D, Globig A, Beer M (2010) (Highly pathogenic) avian influenza as a zoonotic agent. Vet Microbiol 140: 237-245. doi:10.1016/j.vetmic.2009.08.022. PubMed: 19782482. 19782482 10.1016/j.vetmic.2009.08.022

[9] Webster RG, Bean WJ, Gorman OT, Chambers TM, Kawaoka Y (1992) Evolution and ecology of influenza A viruses. Microbiol Mol Biol Rev 56: 152-179. PubMed: 1579108. 10.1128/mr.56.1.152-179.1992 PMC372859 1579108

[10] Scholtissek C, Bürger H, Kistner O, Shortridge KF (1985) The nucleoprotein as a possible major factor in determining host specificity of influenza H3N2 viruses. Virology 147: 287-294. doi:10.1016/0042-6822(85)90131-X. PubMed: 2416114. 2416114 10.1016/0042-6822(85)90131-x

[11] Nelson MI, Vincent AL, Kitikoon P, Holmes EC, Gramer MR (2012) Evolution of Novel Reassortant A/H3N2 Influenza Viruses in North American Swine and Humans, 2009–2011. J Virol 86: 8872-8878. doi:10.1128/JVI.00259-12. PubMed: 22696653. 22696653 10.1128/JVI.00259-12 PMC3421719

[12] Ito T, Couceiro JNSS, Kelm S, Baum LG, Krauss S et al. (1998) Molecular Basis for the Generation in Pigs of Influenza A Viruses with Pandemic Potential. J Virol 72: 7367-7373. PubMed: 9696833. 9696833 10.1128/jvi.72.9.7367-7373.1998 PMC109961

[13] Lu W, Hisatsune A, Koga T, Kato K, Kuwahara I et al. (2006) Cutting edge: enhanced pulmonary clearance of Pseudomonas aeruginosa by Muc1 knockout mice. J Immunol 176: 3890-3894. PubMed: 16547220. 16547220 10.4049/jimmunol.176.7.3890

[14] Nelli RK, Kuchipudi SV, White GA, Perez BB, Dunham SP et al. (2010) Comparative distribution of human and avian type sialic acid influenza receptors in the pig. BMC Veterinary Res 6: 4. doi:10.1186/1746-6148-6-4. PubMed: 20105300. 10.1186/1746-6148-6-4 PMC2832630 20105300

[15] Van Poucke SG, Nicholls JM, Nauwynck HJ, Van Reeth K (2010) Replication of avian, human and swine influenza viruses in porcine respiratory explants and association with sialic acid distribution. Virol J 7: 38. doi:10.1186/1743-422X-7-38. PubMed: 20158900. 20158900 10.1186/1743-422X-7-38 PMC2829537

[16] Trebbien R, Larsen LE, Viuff BM (2011) Distribution of sialic acid receptors and influenza A virus of avian and swine origin in experimentally infected pigs. Virol J 8: 434. doi:10.1186/1743-422X-8-434. PubMed: 21902821. 21902821 10.1186/1743-422X-8-434 PMC3177912

[17] Monzon ME, Fregien N, Schmid N, Falcon NS, Campos M et al. (2010) Reactive oxygen species and hyaluronidase 2 regulate airway epithelial hyaluronan fragmentation. J Biol Chem 285: 26126-26134. doi:10.1074/jbc.M110.135194. PubMed: 20554532. 20554532 10.1074/jbc.M110.135194 PMC2924017

[18] Ehrhardt C, Seyer R, Hrincius ER, Eierhoff T, Wolff T et al. (2010) Interplay between influenza A virus and the innate immune signaling. Microbes Infect 12: 81-87. doi:10.1016/j.micinf.2009.09.007. PubMed: 19782761. 19782761 10.1016/j.micinf.2009.09.007

[19] Fischer BM, Rochelle LG, Voynow JA, Akley NJ, Adler KB (1999) Tumor Necrosis Factor-alpha Stimulates Mucin Secretion and Cyclic GMP Production by Guinea Pig Tracheal Epithelial Cells In Vitro. Am J Respir Cell Mol Biol 20: 413-422. doi:10.1165/ajrcmb.20.3.3393. PubMed: 10030839. 10030839 10.1165/ajrcmb.20.3.3393

[20] Barbé F, Atanasova K (In press) Van Reeth K Cytokines and acute phase proteins associated with acute swine influenza infection in pigs. Vet J, Corrected Proof 10.1016/j.tvjl.2009.12.012 PMC7129392 20097110

[21] Van Reeth K (2000) Cytokines in the pathogenesis of influenza. Vet Microbiol 74: 109-116. doi:10.1016/S0378-1135(00)00171-1. PubMed: 10799783. 10799783 10.1016/s0378-1135(00)00171-1

[22] Hayden FG, Fritz R, Lobo MC, Alvord W, Strober W et al. (1998) Local and systemic cytokine responses during experimental human influenza A virus infection. Relation to symptom formation and host defense. J Clin Invest 101: 643-649. doi:10.1172/JCI1355. PubMed: 9449698. 9449698 10.1172/JCI1355 PMC508608

[23] Uen YH, Lin SR, Wu CH, Hsieh JS, Lu CY et al. (2006) Clinical significance of MUC1 and c-Met RT-PCR detection of circulating tumor cells in patients with gastric carcinoma. Clin Chim Acta 367: 55-61. doi:10.1016/j.cca.2005.11.013. PubMed: 16403482. 16403482 10.1016/j.cca.2005.11.013

[24] Haß J, Matuszewski S, Cieslik D, Haase M (2011) The role of swine as “mixing vessel” for interspecies transmission of the influenza A subtype H1N1: A simultaneous Bayesian inference of phylogeny and ancestral hosts. Infect Genet Evol 11: 437-441. doi:10.1016/j.meegid.2010.12.001. PubMed: 21163369. 21163369 10.1016/j.meegid.2010.12.001

[25] Randall RE, Goodbourn S (2008) Interferons and viruses: an interplay between induction, signalling, antiviral responses and virus countermeasures. J Gen Virol 89: 1-47. doi:10.1099/vir.0.83391-0. PubMed: 18089727. 18089727 10.1099/vir.0.83391-0

[26] Der SD, Zhou A, Williams BRG, Silverman RH (1998) Identification of genes differentially regulated by interferon α, β, or γ using oligonucleotide arrays. Proc Natl Acad Sci USA 95: 15623-15628. doi:10.1073/pnas.95.26.15623. PubMed: 9861020. 9861020 10.1073/pnas.95.26.15623 PMC28094

[27] Vareille M, Kieninger E, Edwards MR, Regamey N (2011) The Airway Epithelium: Soldier in the Fight against Respiratory Viruses. Clin Microbiol Rev 24: 210-229. doi:10.1128/CMR.00014-10. PubMed: 21233513. 21233513 10.1128/CMR.00014-10 PMC3021210

[28] Flores-Ocelotl MdR, Rosas-Murrieta NH, Vallejo-Ruiz V, Reyes-Leyva J, Herrera-Camacho I et al. (2011) Transcription of interferon stimulated genes in response to porcine rubulavirus infection in vitro. Braz J Microbiol 42: 1167-1175. doi:10.1590/S1517-83822011000300041. 24031738 10.1590/S1517-838220110003000041 PMC3768783

[29] Kato H, Takeuchi O, Sato S, Yoneyama M, Yamamoto M et al. (2006) Differential roles of MDA5 and RIG-I helicases in the recognition of RNA viruses. Nature 441: 101-105. doi:10.1038/nature04734. PubMed: 16625202. 16625202 10.1038/nature04734

[30] Ma W, Kahn RE, Richt JA (2009) The pig as a mixing vessel for influenza viruses: human and veterinary implications. Mol Genet Med 3: 158-166. PubMed: 19565018. PMC2702078 19565018

[31] Ma W, Lager KM, Vincent AL, Janke BH, Gramer MR et al. (2009) The Role of Swine in the Generation of Novel Influenza Viruses. Zoonoses & Public Health 56: 326-337 10.1111/j.1863-2378.2008.01217.x 19486316

[32] Jewell NA, Cline T, Mertz SE, Smirnov SV, Flaño E et al. (2010) Lambda Interferon Is the Predominant Interferon Induced by Influenza A Virus Infection In Vivo. J Virol 84: 11515-11522. doi:10.1128/JVI.01703-09. PubMed: 20739515. 20739515 10.1128/JVI.01703-09 PMC2953143

[33] Pensaert M, Ottis K, Vandeputte J, Kaplan MM, Bachmann PA (1981) Evidence for the natural transmission of influneza A virus from wild ducks to swine and its potential importance for man. Bull WHO 59: 75-78. PubMed: 6973418. 6973418 PMC2396022

[34] Zhang H (2009) Tissue and host tropism of influenza viruses: importance of quantitative analysis. Sci China C 52: 1101-1110. doi:10.1007/s11427-009-0161-x. PubMed: 20016966. 10.1007/s11427-009-0161-x 20016966

[35] Mehle A, Dugan VG, Taubenberger JK, Doudna JA (2012) Reassortment and Mutation of the Avian Influenza Virus Polymerase PA Subunit Overcome Species Barriers. J Virol 86: 1750-1757. doi:10.1128/JVI.06203-11. PubMed: 22090127. 22090127 10.1128/JVI.06203-11 PMC3264373

[36] Hayman A, Comely S, Lackenby A, Hartgroves LCS, Goodbourn S et al. (2007) NS1 Proteins of Avian Influenza A Viruses Can Act as Antagonists of the Human Alpha/Beta Interferon Response. J Virol 81: 2318-2327. doi:10.1128/JVI.01856-06. PubMed: 17182679. 17182679 10.1128/JVI.01856-06 PMC1865923

[37] Shapira SD, Gat-Viks I, Shum BO, Dricot A, de Grace MM et al. (2009) A physical and regulatory map of host-influenza interactions reveals pathways in H1N1 infection. Cell 139: 1255-1267. doi:10.1016/j.cell.2009.12.018. PubMed: 20064372. 20064372 10.1016/j.cell.2009.12.018 PMC2892837

[38] Cohen-Daniel L, Zakay-Rones Z, Resnick IB, Shapira MY, Dorozhko M et al. (2009) Emergence of oseltamivir-resistant influenza A/H3N2 virus with altered hemagglutination pattern in a hematopoietic stem cell transplant recipient. J Clin Virol 44: 138-140. doi:10.1016/j.jcv.2008.11.014. PubMed: 19157971. 19157971 10.1016/j.jcv.2008.11.014

[39] Karlas A, Machuy N, Shin Y, Pleissner KP, Artarini A et al. (2010) Genome-wide RNAi screen identifies human host factors crucial for influenza virus replication. Nature 463: 818-822. doi:10.1038/nature08760. PubMed: 20081832. 20081832 10.1038/nature08760

[40] Ciencewicki JM, Brighton LE, Jaspers I (2009) Localization of type I interferon receptor limits interferon-induced TLR3 in epithelial cells. J Interferon Cytokine Res 29: 289-297. doi:10.1089/jir.2008.0075. PubMed: 19231996. 19231996 10.1089/jir.2008.0075 PMC2956593

[41] Mangan NE, Fung KY (2012) Type I interferons in regulation of mucosal immunity. Immunol Cell Biol 90: 510-519. doi:10.1038/icb.2012.13. PubMed: 22430250. 22430250 10.1038/icb.2012.13

[42] Squires K, Watkins A, König K (2010) H1N1 influenza in an extremely premature baby with chronic lung disease. Pediatr Pulmonol 45: 409-410. doi:10.1002/ppul.21177. PubMed: 20232457. 20232457 10.1002/ppul.21177

[43] König R, Stertz S, Zhou Y, Inoue A, Hoffmann HH et al. (2010) Human host factors required for influenza virus replication. Nature 463: 813-817. doi:10.1038/nature08699. PubMed: 20027183. 20027183 10.1038/nature08699 PMC2862546

[44] Vincent AL, Ma W, Lager KM, Janke BH, Richt JA (2008) Swine influenza viruses a North American perspective. Adv Virus Res 72: 127-154. doi:10.1016/S0065-3527(08)00403-X. PubMed: 19081490. 19081490 10.1016/S0065-3527(08)00403-X

[45] Sutejo R, Yeo DS, Myaing MZ, Hui C, Xia J et al. (2012) Activation of Type I and III Interferon Signalling Pathways Occurs in Lung Epithelial Cells Infected with Low Pathogenic Avian Influenza Viruses. PLOS ONE 7: e33732. doi:10.1371/journal.pone.0033732. PubMed: 22470468. 22470468 10.1371/journal.pone.0033732 PMC3312346

[46] Lam WY, Tang JW, Yeung ACM, Chiu LCM, Sung JJY et al. (2008) Avian Influenza Virus A/HK/483/97(H5N1) NS1 Protein Induces Apoptosis in Human Airway Epithelial Cells. J Virol 82: 2741-2751. doi:10.1128/JVI.01712-07. PubMed: 18199656. 18199656 10.1128/JVI.01712-07 PMC2258969

[47] Hale BG, Randall RE, Ortín J, Jackson D (2008) The multifunctional NS1 protein of influenza A viruses. J Gen Virol 89: 2359-2376. doi:10.1099/vir.0.2008/004606-0. PubMed: 18796704. 18796704 10.1099/vir.0.2008/004606-0

[48] Schmolke M, García-Sastre A (2010) Evasion of innate and adaptive immune responses by influenza A virus. Cell Microbiol 12: 873-880. doi:10.1111/j.1462-5822.2010.01475.x. PubMed: 20482552. 20482552 10.1111/j.1462-5822.2010.01475.x PMC2897956

[49] De Vleeschauwer A, Atanasova K, Van Borm S, Berg T, Rasmussen TB et al. (2009) Comparative pathogenesis of an avian H5N2 and a swine H1N1 influenza virus in pigs. PLOS ONE 4: e6662. doi:10.1371/journal.pone.0006662. PubMed: 19684857. 19684857 10.1371/journal.pone.0006662 PMC2722722

[50] Beare AS, Webster RG (1991) Replication of avian influenza viruses in humans. Arch Virol 119: 37-42. doi:10.1007/BF01314321. PubMed: 1863223. 1863223 10.1007/BF01314321

[51] Alexopoulou L, Holt AC, Medzhitov R, Flavell RA (2001) Recognition of double-stranded RNA and activation of NF-[kappa]B by Toll-like receptor 3. Nature 413: 732-738. doi:10.1038/35099560. PubMed: 11607032. 11607032 10.1038/35099560

[52] Manuse MJ, Parks GD (2010) TLR3-dependent upregulation of RIG-I leads to enhanced cytokine production from cells infected with the parainfluenza virus SV5. Virology 397: 231-241. doi:10.1016/j.virol.2009.11.014. PubMed: 19948350. 19948350 10.1016/j.virol.2009.11.014 PMC2813885

[53] Yoneyama M, Kikuchi M, Natsukawa T, Shinobu N, Imaizumi T et al. (2004) The RNA helicase RIG-I has an essential function in double-stranded RNA-induced innate antiviral responses. Nat Immunol 5: 730-737. doi:10.1038/ni1087. PubMed: 15208624. 15208624 10.1038/ni1087

[54] Nazmi A, Dutta K, Basu A (2011) RIG-I Mediates Innate Immune Response in Mouse Neurons Following Japanese Encephalitis Virus Infection. PLOS ONE 6: e21761. doi:10.1371/journal.pone.0021761. PubMed: 21738791. 21738791 10.1371/journal.pone.0021761 PMC3128083

[55] Nasirudeen AMA, Wong HH, Thien P, Xu S, Lam K-P et al. (2011) RIG-I, MDA5 and TLR3 Synergistically Play an Important Role in Restriction of Dengue Virus Infection. PLOS Negl Trop Dis 5: e926. doi:10.1371/journal.pntd.0000926. PubMed: 21245912. 21245912 10.1371/journal.pntd.0000926 PMC3014945

[56] Hui KPY, Lee SMY, Cheung C-y, Mao H, Lai AKW et al. (2011) H5N1 Influenza Virus–Induced Mediators Upregulate RIG-I in Uninfected Cells by Paracrine Effects Contributing to Amplified Cytokine Cascades. J Infect Dis 204: 1866-1878. doi:10.1093/infdis/jir665. PubMed: 22013225. 22013225 10.1093/infdis/jir665

[57] Haye K, Burmakina S, Moran T, García-Sastre A, Fernandez-Sesma A (2009) The NS1 Protein of a Human Influenza Virus Inhibits Type I Interferon Production and the Induction of Antiviral Responses in Primary Human Dendritic and Respiratory Epithelial Cells. J Virol 83: 6849-6862. doi:10.1128/JVI.02323-08. PubMed: 19403682. 19403682 10.1128/JVI.02323-08 PMC2698524

[58] Honda K, Taniguchi T (2006) IRFs: master regulators of signalling by Toll-like receptors and cytosolic pattern-recognition receptors. Nat Rev Immunol 6: 644-658. doi:10.1038/nri1900. PubMed: 16932750. 16932750 10.1038/nri1900

[59] Nelli RK, Dunham SP, Kuchipudi SV, White GA, Baquero-Perez B et al. (2012) Mammalian Innate Resistance to Highly Pathogenic Avian Influenza H5N1 Virus Infection Is Mediated through Reduced Proinflammation and Infectious Virus Release. J Virol 86: 9201-9210. doi:10.1128/JVI.00244-12. PubMed: 22718824. 22718824 10.1128/JVI.00244-12 PMC3416141

