# Supplementary figures and images for: Antiviral Responses by Swine Primary Bronchoepithelial Cells Are Limited Compared to Human Bronchoepithelial Cells Following Influenza Virus Infection
Source: PLoS One. 2013 Jul 10;8(7):e70251. doi: 10.1371/journal.pone.0070251 (PMC3707852; doi:10.1371/journal.pone.0070251)

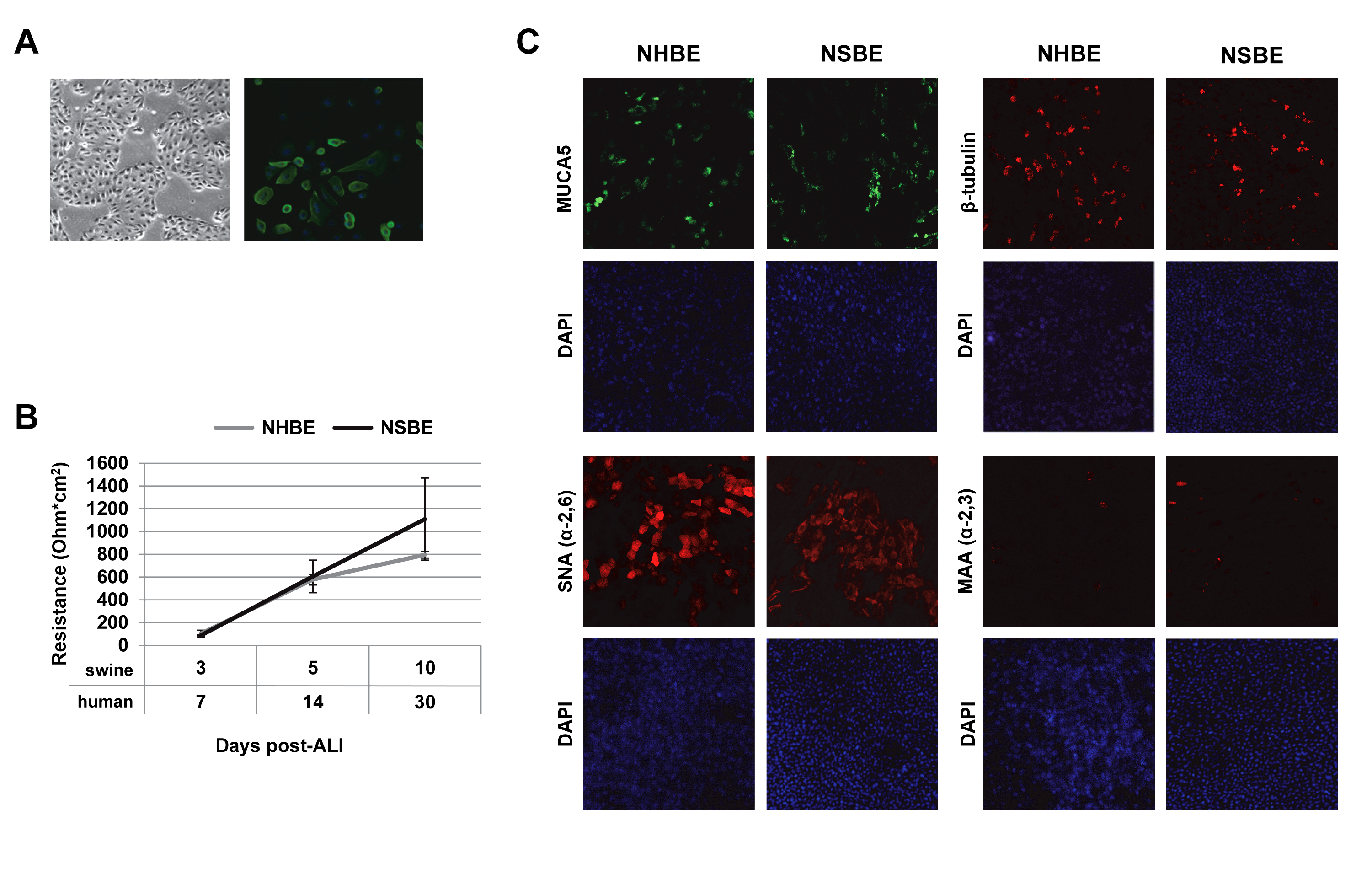

Supplement: Figure S1 — (A) Phase micrograph of primary swine cells (right) and fluorescent microscopy (left) showing cytokeratin staining (green) and DAPI (blue). (B) Trans-epithelial resistance of differentiated human and swine cells was measured over the indicated times post air-liquid interface (ALI). (C) Differentiated NHBE and NSBE cells were immune stained to show composition of goblet cells (top left), ciliated cells (top right), α-2,6 linked sialic acid (bottom left) and α-2,3 linked sialic acid (bottom right) expression. Cells were counterstained with DAPI (blue). (TIF) [file pone.0070251.s001.tif]
